# Special Issue “Biomechanical Analysis in Physical Activity and Sports—2nd Edition”

**DOI:** 10.3390/jfmk11020132

**Published:** 2026-03-24

**Authors:** Pedro Forte

**Affiliations:** 1 Department of Sports, Higher Institute of Educational Sciences of the Douro, 4560-708 Penafiel, Portugal; pedromiguel.forte@iscedouro.pt; 2 Department of Sports Sciences, Instituto Politécnico de Bragança, 5300-253 Bragança, Portugal; 3 Research Center for Active Living and Wellbeing, Instituto Politécnico de Bragança, 5300-253 Bragança, Portugal

## 1. Introduction

Biomechanics continues to represent a fundamental pillar for understanding human movement, athletic performance, injury mechanisms, and rehabilitation processes [1,2,3,4]. Beyond its descriptive capacity, biomechanics provides a critical framework for interpreting how mechanical and neuromuscular factors interact in performance and clinical contexts. In recent years, technological developments have significantly enhanced our ability to quantify these processes with higher precision, particularly regarding neuromuscular function, joint kinetics, and dynamic outputs such as ground reaction forces [2,5,6,7,8,9].

This progress has been largely driven by the emergence of markerless motion capture systems, wearable sensors, inertial measurement units, and advances in computer vision. These technologies have not only increased measurement resolution but also expanded the contexts in which biomechanical data can be collected, moving beyond controlled laboratory environments to real-world settings [6,7,8,9]. As a result, biomechanics is progressively transitioning from a laboratory-centered discipline toward a more applied and ecologically grounded science.

Importantly, this shift has been accompanied by a growing recognition of the need for ecological validity. The demand is high for research that bridges the gap between laboratory findings and real-world applications in sport and clinical practice. This includes the development of methodologies capable of capturing performance and movement behavior in natural environments, while maintaining acceptable levels of accuracy and reliability [2,10,11,12,13,14]. Such approaches are essential to ensure that biomechanical insights are not only scientifically robust but also practically meaningful.

At the same time, advances in data acquisition have enabled more comprehensive assessments of neuromuscular and mechanical variables. The integration of electromyography with kinematic and kinetic data, alongside the estimation of joint moments and ground reaction forces using wearable and vision-based systems, reflects a trend toward multimodal analysis [2,5,6,7,8,9]. Artificial intelligence and machine learning techniques are increasingly being incorporated to process and interpret these complex datasets, further enhancing analytical capabilities. However, these developments also introduced challenges, particularly regarding data synchronization, validation against gold standard systems, and the influence of environmental variability [5,6,7].

The balance between accuracy and ecological validity remains a central issue in contemporary biomechanics. Although laboratory-based systems continue to represent the gold standard, their limited applicability in real-world contexts has driven the adoption of portable and field-based solutions. The current evidence suggests that hybrid approaches, combining laboratory rigor with field-based data collection, may offer a viable pathway to address this challenge [4,7,8]. Such approaches allow researchers and practitioners to retain methodological robustness while increasing the ecological relevance of their findings.

From a practical perspective, these developments have important implications for decision-making in sport and clinical settings. The use of tiered monitoring strategies, integrating different levels of technological complexity, may facilitate the implementation of biomechanics in applied environments. Additionally, the need is increasing for transparent and interpretable analytical models, particularly when machine learning is used to inform performance optimization or injury risk assessment [10,11,12,13,14,15,16].

Looking forward, several priorities should be considered. The establishment of standardized validation protocols remains essential for ensuring comparability across studies and technologies. Furthermore, the development of large-scale, multi-site datasets will be critical to increase the generalizability of findings. Emerging tools, such as extended reality, also offer promising opportunities to simulate sport-specific scenarios while maintaining experimental control [10,11,12,13,15].

Despite these advances, limitations persist. These include gaps in sensor validation, challenges in capturing sport-specific contexts, and concerns regarding the generalizability of machine learning models trained on limited datasets [15,16,17]. Consequently, caution is required when interpreting field-based data, particularly in the absence of robust validation across different populations and settings. In this context, interdisciplinary collaboration between biomechanists, sport scientists, engineers, and clinicians will be essential for fully exploiting the potential of current and emerging technologies [6,7,8,9].

Overall, biomechanics is undergoing an evolution toward more applied, integrative, and ecologically valid approaches. The challenge moving forward will be maintaining scientific rigor while ensuring that research outcomes remain relevant and transferable to real-world performance and clinical practice. The present Special Issue, “Biomechanical Analysis in Physical Activity and Sports—2nd Edition”, reflects this evolution. The published studies collectively demonstrate methodological innovation, applied sport relevance, and clinical translation, also addressing under-represented populations such as female athletes, older adults, and African recreational runners.

## 2. Recent Developments in Sports Biomechanics

Advances in myotonometry allow the non-invasive quantification of the mechanical properties of muscle, providing new opportunities to monitor neuromuscular function in sport and clinical contexts. Parameters such as stiffness, elasticity, and relaxation time offer insights into the mechanical behavior of muscle tissue and its response to different types of contraction and training stimuli. These measurements may help detect fatigue-related changes, guide recovery strategies, and support individualized load management. By complementing traditional strength assessments with mechanical muscle profiling, myotonometry contributes to a more comprehensive understanding of how neuromuscular systems adapt to exercise and training demands.

Jump performance remains a central indicator of lower limb power and neuromuscular readiness, yet its biomechanical interpretation increasingly requires the consideration of contextual constraints. In sport environments, jumping tasks are often performed under conditions that involve additional technical, perceptual, or tactical demands, which can influence movement coordination and force production strategies. External constraints such as ball handling, timing requirements, or interaction with opponents may modify joint mechanics and body alignment, meaning that mechanical variables alone do not fully explain performance outcomes. Recognizing the interaction between biomechanical and contextual factors is therefore essential for developing more ecologically valid models of sport performance.

Several contributions in this Special Issue addressed strength and resistance exercise from a biomechanical perspective, highlighting the complex interaction between muscular capacity and the mechanical constraints imposed by different exercises. Movements such as squats, pulldowns, and high-intensity functional tasks demonstrate that force production is influenced by not only maximal strength but also technique, joint positioning, and leverage throughout the movement. These constraints can create mechanically challenging phases within an exercise, requiring coordinated neuromuscular strategies to overcome them. Understanding these interactions is important for refining exercise prescription, optimizing training adaptations, and increasing the efficiency and safety of resistance training programs.

Lower limb injury risk was a recurring theme across the studies included in this Special Issue, reflecting the central role of biomechanical analysis in injury prevention. Mechanical factors such as joint loading, muscle strength balance, and movement coordination can influence the stresses experienced by tissues during athletic activity. Additionally, injury risk is shaped by population-specific characteristics including body composition, anthropometric features, training background, and cultural or environmental contexts. Investigating these variables across diverse populations contributes to a more comprehensive understanding of how movement mechanics interact with individual characteristics, ultimately supporting the development of more targeted injury prevention strategies.

Accurate biomechanical assessment requires reliable measurement tools and standardized testing procedures. As new technologies continue to expand the possibilities for measuring human movement, ensuring consistency between devices and protocols remains an important methodological challenge. Differences in measurement systems, data processing algorithms, and testing conditions can influence the obtained values of biomechanical variables. Consequently, careful validation and methodological transparency are essential to guarantee that results are comparable across studies and applicable in practical settings. Strengthening reliability and standardization will remain a key step in advancing the scientific rigor and practical relevance of biomechanics research.

Rehabilitation and clinical translation represent important applications of biomechanical research, particularly when movement analysis is integrated with functional tasks that reflect everyday activities. Assessing movements such as sit-to-stand transitions, gait, or core stabilization exercises provides valuable insights into how neuromuscular control evolves during recovery from injury or dysfunction. These analyses allow clinicians to identify compensatory strategies, monitor functional improvements, and design more targeted rehabilitation interventions. As biomechanical technologies become more accessible, their integration into clinical practice has the potential to enhance evidence-based rehabilitation and support the restoration of safe and efficient movement patterns.

## 3. Knowledge Gaps

Despite the significant advances presented in this Special Issue, several limitations remain within the current body of literature. Many studies continue to rely on relatively small and homogeneous samples, restricting the generalizability of findings across competitive levels, sexes, age groups, and cultural contexts. Moreover, biomechanical variables alone frequently explain only a portion of performance variability, reinforcing the need to integrate physiological, psychological, and environmental determinants. Longitudinal and intervention-based designs are still comparatively scarce, limiting causal inference and the translation of findings into training and rehabilitation practice. In addition, device validation and cross-standardization across dynamometers, motion capture systems, and wearable technologies require further refinement to ensure methodological consistency. Notably, female athletes, older adults, and ethnically diverse populations remain under-represented, highlighting persistent demographic imbalances in sports biomechanics research.

## 4. Future Research Directions

Future investigations should therefore adopt large-scale, multicenter longitudinal approaches that integrate biomechanical, physiological, perceptual–cognitive, and contextual variables within unified analytical frameworks. The development of multimodal assessment models combining electromyography, myotonometry, kinematics, kinetics, wearable sensors, and machine learning algorithms may enable more comprehensive and individualized performance profiling. Increased emphasis on sex- and age-specific research—accounting for hormonal influences, aging-related neuromuscular changes, and population-specific morphology—is needed. Ecologically valid, sport-specific interventions that incorporate perceptual and cognitive demands should be prioritized to enhance the transferability of research findings to competitive environments. Furthermore, culturally informed footwear design, refined load distribution models, and optimized rehabilitation strategies during immobilization and disuse deserve systematic investigation. Ultimately, integrative approaches that bridge laboratory precision with applied sport settings will be essential for advancing injury prevention, performance optimization, and evidence-based rehabilitation.

## 5. Conclusions

The studies published in this Special Issue collectively advance contemporary biomechanical research by expanding methodological approaches, enhancing clinical translation, and incorporating diverse populations. From muscle viscoelastic adaptations and jump mechanics to injury risk indicators and rehabilitation protocols, the contributions reflect the multidimensional nature of biomechanics in physical activity and sports.

Continued interdisciplinary collaboration and technological integration will be fundamental to translating biomechanical knowledge into optimized training strategies, injury prevention frameworks, and evidence-based rehabilitation programs.
